# Diabetes mellitus is associated with increased mortality during tuberculosis treatment: a prospective cohort study among tuberculosis patients in South-Eastern Amahra Region, Ethiopia

**DOI:** 10.1186/s40249-016-0115-z

**Published:** 2016-03-21

**Authors:** Mahteme Haile Workneh, Gunnar Aksel Bjune, Solomon Abebe Yimer

**Affiliations:** Institute of Health and Society, Faculty of Medicine, University of Oslo, Oslo, Norway; Amhara Regional State Health Bureau, Bahir-Dar, Ethiopia; Department of Microbiology, Oslo University Hospital, Oslo, Norway; Department of Bacteriology and Immunology, Norwegian Institute of Public Health, Oslo, Norway

**Keywords:** Tuberculosis, Diabetes mellitus, Association, Symptoms, Treatment outcome, Amhara Region, Ethiopia

## Abstract

**Background:**

There is growing evidence suggesting that diabetes mellitus (DM) affects disease presentation and treatment outcome in tuberculosis (TB) patients. This study aimed at investigating the role of DM on clinical presentations and treatment outcomes among newly diagnosed TB patients.

**Methods:**

A prospective cohort study was conducted in South-Eastern Amhara Region, Ethiopia from September 2013 till March 2015. Study subjects were consecutively recruited from 44 randomly selected health facilities in the study area. Participants were categorized into two patient groups, namely, patients with TB and DM (TBDM) and TB patients without DM (TBNDM). Findings on clinical presentations and treatment outcomes were compared between the two patient groups. Cox proportional hazard regression analysis was applied to identify factors associated with death.

**Results:**

Out of 1314 TB patients enrolled in the study, 109 (8.3 %) had coexisting DM. TBDM comorbidity [adjusted hazard ratio (AHR) 3.96; 95 % confidence interval (C.I.) (1.76–8.89)], and TB coinfection with human immunodeficiency virus (HIV) [AHR 2.59; 95 % C.I. (1.21–5.59)] were associated with increased death. TBDM and TBNDM patients did not show significant difference in clinical symptoms at baseline and during anti-TB treatment period. However, at the 2^nd^ month of treatment, TBDM patients were more symptomatic compared to patients in the TBNDM group.

**Conclusions:**

The study showed that DM is associated with increased death during TB treatment. DM has no association with clinical presentation of TB except at the end of the intensive phase treatment. Routine screening of TB patients for DM is recommended for early diagnosis and treatment of patients with TBDM comorbidity.

**Electronic supplementary material:**

The online version of this article (doi:10.1186/s40249-016-0115-z) contains supplementary material, which is available to authorized users.

## Multilingual abstracts

Please see Additional file 1 for translations of the abstract into the six official working languages of the United Nations.

## Background

Tuberculosis (TB) is a major public health threat in the developing world [1]. It is clear that the current TB control strategy has reduced the TB incidence [1, 2]. However, there are more TB cases today than at any other time in history [3]. This is because of the emergence of multidrug-resistant (MDR) TB strains, human immunodeficiency virus (HIV) epidemic [3] and other risk factors such as smoking, diabetes mellitus (DM), malnutrition, alcohol abuse, indoor air pollution, malignancies and an aging population [4].

DM has recently shown an alarming increase worldwide and there is growing evidence indicating that DM affects TB disease presentation and treatment outcome [5, 6]. A number of studies showed higher frequencies of certain clinical findings such as lower lung field lesions, cavities and acid-fast bacilli (AFB) smear positivity among patients with TB and DM (TBDM) comorbidity [7–10]. Adverse effects of DM on TB treatment outcomes, i.e. increased risk of death, treatment failure, default and relapse were reported in many studies [11, 12]. Few studies reported small or no difference in clinical presentations and treatment outcomes between TBDM and TB without DM (TBNDM) patient groups [13, 14].

Sub-Saharan African countries which are the epicenter for TB and HIV infection are currently affected by the growing burden of DM. The interaction of these diseases is conspiring to increase the negative impacts of all three conditions in sub-Saharan Africa more than any other region [15]. Ethiopia is one of the high TB burden countries in sub-Saharan Africa. According to the World Health Organization (WHO) report, the country has currently achieved the 2015 Millennium Development Goal (MDG) targets of reducing cases and deaths from TB [16]. However, the growing challenges of MDR-TB and TB/HIV coinfection are affecting the TB control efforts [16, 17]. Recent studies in Ethiopia reported that DM is contributing to increased TB burden. Few studies conducted in the Amhara Region of Ethiopia showed high prevalence of DM among TB patients [18, 19]. Nonetheless, no study to date has investigated the association of DM with clinical presentations and treatment outcomes of TB in the region. Thus, this study was conducted to explore the role of DM on clinical presentations and treatment outcomes of newly diagnosed TB patients.

## Methods

### Study area and setting

The study was conducted in South-Eastern part of Amhara Region, Ethiopia. South-Eastern Amhara Region consists of four zones and one City Administration, namely, North Wollo, South Wollo, North Shewa, Oromia Special Zone and Dessie City Administration. The total population is estimated at 7,358,301, and of these were 3,684,735 men and 3,673,566 women [20].

### Study design and population

A prospective cohort study was conducted in 44 randomly selected health facilities (HFs) in the study area. The study population included all newly diagnosed TB patients aged 15 years and above who were consecutively registered for treatment at the directly observed treatment short course (DOTS) units. The study was conducted from September 2013 till March 2015. Study subjects were categorized into TBDM and TBNDM patient groups. Each patient group was followed from enrollment until completion of anti-TB treatment.

### Sampling method and sample size

Random sampling technique was applied to select study sites. There were a total of 420 HFs (326 (78 %) government and 94 (22 %) private HFs) in the study area, of these, only 102 (31 %) government and 20 (21 %) private HFs were eligible to provide TB, HIV and DM diagnostic and treatment services. Among the 20 private HFs, only 5 (25 %) provided TB, HIV and DM services in a continuous manner. Fifteen (75 %) of the private HFs encountered frequent TB service interruption for various reasons and were excluded from the study. Finally, out of the 102 (31 %) government HFs eligible for the study, 39 (38 %) HFs were randomly selected. By adding the five private HFs that were continuously providing TB, HIV and DM services in the study area, a total of 44 HFs were selected as study sites.

Sample size was calculated using the standard formula for estimating single population proportion, (n) = {[z^2^*p (1-p)]/d^2^}. We considered 95 % confidence interval (CI) and a margin error of 3 %. As there were no previous similar studies in the study area, we also assumed that 50 % of the TB patients would have difference in clinical presentations and treatment outcomes. Based on these assumptions, the minimum sample size required for the study was estimated at 1067. By adding 10 % for non-response, the total sample size was calculated to be 1174 patients. However, we included all of the 1335 patients that reported to the study sites during the study period. This amounted to 113 % of the minimum sample size required for the study.

### Inclusion and exclusion criteria

Newly diagnosed TB patients aged 15 years and above, known DM patients who were newly diagnosed for TB, newly diagnosed TB patients who at the initiation of TB treatment were negative for DM investigation but developed DM during the course of ant-TB treatment, transferred-in TB patients who had never started treatment in the transferring out HFs, and newly diagnosed TB patients who started and completed their treatment in the selected study sites and satellite health posts were included in the study, whereas TB patients less than 15 years of age, retreatment cases, patients who could not offer informed consent, known or suspected MDR-TB cases, patients with malignancy and patients who were on immunosuppressive therapy were excluded from the study.

TB diagnosis, disease classification, treatment protocol and treatment outcome evaluations were defined according to the national and WHO guideline [17, 21]. DM was diagnosed by performing screening tests at least two times by random blood glucose (RBS) and/or fasting blood glucose (FBS) tests in accordance with the WHO diagnostic criteria [22]. In addition, self-reporting of DM was used to document previous history of DM. Ziehl-Neelsen staining technique was applied for the detection of AFB by microscopy. All patient groups were treated based on standardized anti-TB treatment regimen regardless of their DM status [17, 21]. Study participants were prospectively followed for 6 months until treatment outcomes were evaluated.

Patients’ responses to treatment were assessed by clinical assessment of sign and symptoms, body weight measurement and follow-up of sputum smear examination results at the end of 2^nd^, 5^th^and 6^th^ months of anti-TB treatment period. If patient’s sputum was positive for AFB at the end of the 2^nd^ month of treatment, sputum smear examination was repeated after 1 month. When a patient remained positive at the end of the 3^rd^ month of treatment, sputum was taken for culture and drug susceptibility testing. Treatment outcomes were classified as cured, treatment completed, death, treatment failure and defaulter [17, 21]. In this analysis, cure and treatment completed were categorized as successful treatment outcomes, whereas death, treatment failure and defaulter were considered as unsuccessful treatment outcomes*.* Provider initiated counseling and testing (PICT) service was provided to screen patients for HIV.

### Operational definitions of variables

Uneducated: study participants who had no formal schooling.

Educated: study participants who attained formal schooling.

Good adherence to anti-TB treatment: patients who took ≥95 % of the prescribed anti-TB regimens.

Good adherence to DM therapy: the extent to which a DM patient practices health worker’s advices (i.e. advises about taking medications, diet, physical activity and attending follow-up visits in DOTS units) which corresponds to implementing 100 % of the recommendations from a health care provider.

### Data collection and quality control

Health workers who were responsible for TB patient care at DOTS units, laboratory technologists, and clinicians responsible for diagnosis, treatment as well as follow-up of TB and DM patients were trained and assigned as data collectors at each study site. Socio-demographic and clinical characteristics of participants were collected using pre-tested semi-structured questionnaire. Clinical and bacteriological characteristics of participants were documented at baseline and during follow-up periods. Data quality was assured by trained supervisors and the principal investigator who regularly checked for data completeness. Blood glucose measurement and weight scale were calibrated before measuring patient’s blood glucose level and body weight. AFB results were checked by internal and external quality control methods.

### Ethical approval

Ethical approval was obtained from the Regional Committee for Research Ethics in Norway (REC-Øst, Norway) (Reference 2013/829/REK sør-øst dated: 05.06.2013) and Ethiopian Science and Technology Ministry (Reference 3.10/355106 dated: 08/01/06). Ethical approval was also secured from health authorities of the study area before the initiation of the study. Patients were informed about the nature of the study and written consent was solicited prior to their participation in the study. Parents/guardians gave informed consent for patients aged 15 to 17 years. TB patients who were found to have DM, MDR-TB and HIV were linked to DM clinics, MDR-TB treatment centers and anti-retroviral therapy (ART) clinics, respectively for further investigation and management.

### Statistical analysis

Data were entered, cleaned and analyzed using Statistical Package for Social Science (SPSS) version 22 Armonk, New York 10504 IBM Corp. software. The original questionnaire was referred and errors corrected by re-entry when inconsistencies occurred. Chi-square test and Fisher’s exact test were used to compare categorical variables as appropriate. Student’s *t* test was applied to compare means for normally distributed variables. Socio-demographic, clinical, and bacteriological parameters were compared between TBDM and TBNDM patient groups. Clinical improvements in both patient groups were measured by assessing decrease in the frequency of clinical symptoms, increases in body mass index (BMI) and changes in sputum smear result during treatment follow-up periods. Kaplan-Meier plot was used to present time to sputum smear conversion and death. Cox proportional hazards regression analysis was performed to investigate factors associated with sputum smear conversion and death. A p-value of ≤ 0.05 was considered statistically significant.

## Results

### Socio-demographic characteristics of participants

A total of 1335 patients consented to participate in the study. Twenty one patients were excluded from the analysis because of non-conclusive DM results. One hundred and nine (8.3 %) patients had coexisting DM. Majority, 642 (53.3 %) TBNDM patients were males and 59 (54.1 %) TBDM patients were females. The means and standard deviations (SD) of participants’ age were 35.0 (±15.0) years for TBNDM and 43.7 (±15.3) years for TBDM patients (*p* < 0.001) (Table 1).

**Table 1:** Socio-demographic characteristics of the study participants, South-Eastern Amhara Region, Ethiopia, September 2013

Type of patients (*n* = 1314)				
Variables	All TB n (%)	TBNDM n (%)	TBDM n (%)	*P*-value
Sex				
Male	692 (52.7)	642 (53.3)	50 (45.9)	
Female	622 (47.3)	563 (46.7)	59 (54.1)	0.14
Age in years				
15–40	928 (70.6)	877 (72.8)	51 (46.8)	<0.001
41–64	285 (21.7)	240 (19.9)	45 (51.3)	
65–89	101 (7.7)	88 (7.3)	13 (11.9)	
Mean (± SD)	35.74 (±15.2)	35.02 (±15.0)	43.73 (±15.3)	<0.001
Marital status				
Single	395 (30.1)	373 (31.0)	22 (20.2)	
Married	919 (69.9)	832 (69.0)	87 (79.8)	0.02
Religion				
Christian	548 (11.7)	508 (42.2)	40 (36.7)	
Muslim	766 (58.3)	697 (57.8)	69 (63.3)	0.27
Residence				
Rural	488 (37.1)	438 (36.3)	50 (45.9)	
Urban	826 (62.9)	767 (63.7)	59 (54.1)	0.05
Education				
Uneducated	618 (47.0)	558 (46.3)	60 (55.0)	
Educated	696 (53.0)	647 (53.7)	49 (45.0)	0.08
Occupation				
Unemployed	376 (28.6)	340 (28.2)	36 (33.0)	
Student	112 (8.5)	106 (8.8)	6 (5.5)	
Self-employed	725 (55.2)	672 (55.8)	53 (48.6)	
Government employed	101 (7.7)	87 (7.2)	14 (12.8)	0.07
Monthly income (USD)^a^				
No income	167 (12.7)	156 (12.9)	11 (10.1)	
≤ 18.9	506 (38.5)	465 (38.6)	41 (37.6)	
19–37.9	303 (23.1)	277 (23.0)	26 (23.9)	
≥ 38	338 (25.7)	307 (25.5)	31 (28.4)	0.79

*TB*: tuberculosis, *TBNDM*: TB without DM, *TBDM*: TB and DM, *SD*: standared deviation, *USD*: United State Dollar

^a^1USD = 21.1 Ethiopian birr

### Clinical profile of study subjects

Seven hundred (58.1 %) TBNDM and 70 (64.2 %) TBDM patients were pulmonary TB (PTB) cases. Three hundred twenty two (46.0 %) TBNDM and 28 (40.0 %) TBDM study participants were smear-positive cases. TB lymphadenitis was the most common form of extra pulmonary TB (EPTB) observed in 237 (51.0 %) TBNDM and 13 (37.1 %) TBDM patients (Table 2).

**Table 2:** Clinical profile of study participants, South-Eastern Amhara Region, Ethiopia, September 2013

Type of patients				
Characteristics	All TB n (%)	TBNDM n (%)	TBDM n (%)	*P-*value
Type of TB (*n* = 1314)				
PTB	770 (58.6)	700 (58.1)	70 (64.2)	
EPTB	544 (41.4)	505 (41.9)	39 (35.8)	0.21
Sputum smear status at baseline (*n* = 770)				
Negative	420 (54.5)	378 (54.0)	42 (60.0)	
Positive	350 (45.5)	322 (46.0)	28 (40.0)	0.33
Type of EPTB (*n* = 500)				
TB lymphadenitis	250 (50.0)	237 (51.0)	13 (37.1)	0.30*
TB spondylitis	39 (7.8)	36 (7.7)	3 (8.6)	
TB peritonitis	36 (7.2)	35 (7.5)	1 (2.9)	
TB enteritis	23 (4.6)	20 (4.3)	3 (8.6)	
Bone TB	18 (3.6)	17 (3.6)	1 (2.9)	
TB abscess	18 (3.6)	16 (3.4)	2 (5.7)	
Others	116 (23.2)	104 (22.4)	12 (34.3)	
HIV status (*n* = 1306)				
Negative	1045 (80.0)	958 (80.0)	87 (79.8)	
Positive	261 (20.0)	239 (20.0)	22 (20.2)	0.96
HIV therapy (*n* = 261)				
ART	155 (59.4)	143 (59.8)	12 (54.5)	
Pre-ART	106 (40.6)	96 (40.2)	10 (45.5)	0.63

*TB*: tuberculosis, *TBNDM*: TB without DM, *TBDM:* TB and DM, *EPTB*: extra pulmonary TB, *PTB*: pulmonary TB, *HIV*: human immuno deficiency virus, *ART*: anti-retroviral

*Fisher’s exact test

### Clinical presentation and sputum smear conversion

**Fig. 1:**
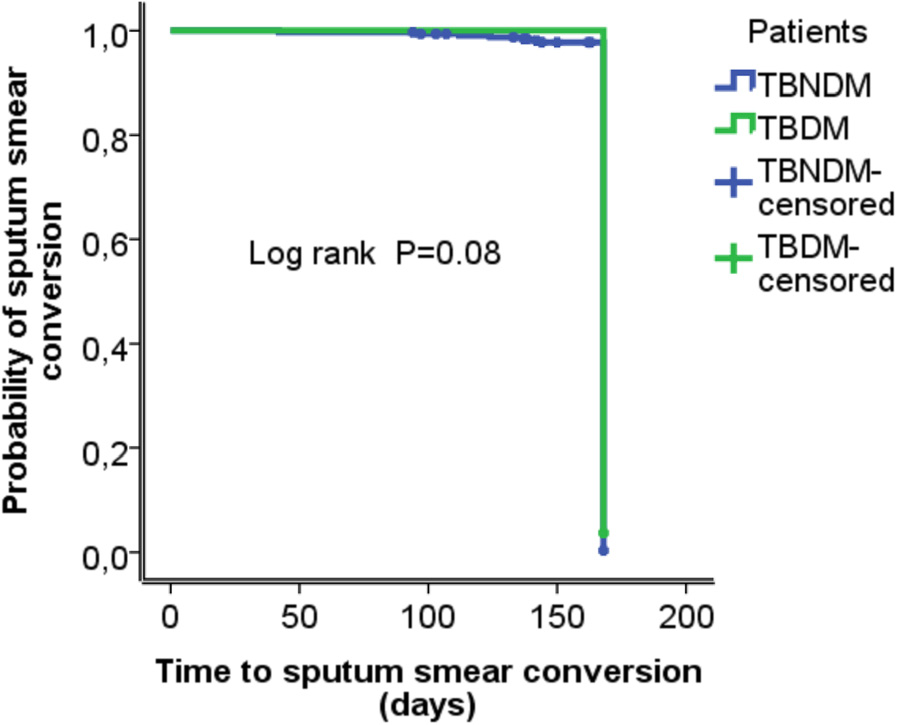
Kaplan-Meier curves for sputum smear conversion comparing TBDM Vs. TBNDM patient groups

At baseline, there was a high frequency of cough 79 (72.5 %) and weight loss 94 (86.2 %) among patients in the TBDM group compared to 784 (65.1 %) patients with cough and 949 (78.8 %) patients with weight loss in the TBNDM group. At the end of 2^nd^ month of anti-TB treatment, 23 (23.0 %) TBDM patients had cough compared to 166 (14.0 %) patients with cough in the TBNDM group (*p* = 0.02). At 5^th^ month, 8 (8.6 %) patients in the TBDM category had a BMI value of ≥25 kg/m^2^ compared to 42 (3.6 %) patients with BMI of ≥25 kg/m^2^ in the TBNDM group (*p =* 0.05). At 6^th^ month treatment period, 9 (9.7 %) patients in the TBDM group had a BMI of ≥25 kg/m^2^ compared to 46 (4.0 %) patients with a BMI value of ≥25 kg/m^2^ in the TBNDM group (*p* = 0.04) (Table 3). Sputum smear conversion was observed in 262 (82.4 %) TBNDM and 22 (81.5 %) TBDM patients at the end of 2^nd^ month treatment period (Fig. 1) (Table 4). Good patient adherence to anti-TB treatment was associated with good sputum conversion [adjusted hazard ratio (AHR) 3.13; 95 % confidence interval (C.I.), (1.34–7.32)] (Table 5).

**Table 3:** Clinical characteristics of study subjects at baseline, 2^nd^, 5^th^ and 6^th^ months of anti-TB treatment period, September 2013- March 2015

Months of anti-TB therapy												
Clinical symptoms	Baseline (*n* = 1314)^a^			2^nd^ month (*n* = 1283)^a^			5^th^ month (*n* = 1246)^a^			6^th^ month (*n* = 1229)^a^		
	TBNDM n (%)	TBDM n (%)	*P*-value	TBNDM n (%)	TBDM n (%)	*P*-value	TBNDM n (%)	TBDM n (%)	*P*-value	TBNDM n (%)	TBDM n (%)	*P*-value
Cough	784 (65.1)	79 (72.5)	0.12	166 (14.0)	23 (23.0)	0.02	24 (2.1)	2 (2.2)	1.00*	18 (1.6)	2 (2.2)	0.66*
Fever	933 (78.4)	80 (73.4)	0.34	166 (14.0)	18 (18.0)	0.28	29 (2.5)	2 (2.2)	1.00*	13 (1.1)	1 (1.1)	1.00*
Hemoptysis	228 (18.9)	24 (22.0)	0.43	18 (1.5)	6 (6.0)	0.008	4 (0.3)	-	-	1 (0.1)	-	-
Weight loss	949 (78.8)	94 (86.2)	0.06	86 (7.3)	14 (14.0)	0.02	14 (1.2)	2 (2.2)	0.34*	10 (0.9)	1 (1.1)	0.58*
Poor appetite	922 (76.5)	86 (78.9)	0.57	154 (13.0)	16 (16.0)	0.39	25 (2.2)	2 (2.2)	1.00*	10 (0.9)	-	-
Night sweat	818 (67.9)	75 (68.8)	0.84	121 (10.2)	6 (6.0)	0.17	21 (1.8)	2 (2.2)	0.69*	14 (1.2)	2 (2.2)	0.34*
Dyspnea	587 (48.7)	52 (47.7)	0.84	93 (7.9)	9 (9.0)	0.68	24 (2.1)	2 (2.2)	1.00*	9 (0.8)	2 (2.2)	0.20*
Chest pain	683 (56.7)	64 (58.7)	0.68	108 (9.1)	9 (9.0)	0.96	26 (2.3)	4 (4.3)	0.27*	9 (0.8)	2 (2.2)	0.20*
Fatigue	967 (80.2)	90 (82.6)	0.56	207 (17.5)	29 (29.0)	0.004	46 (4.0)	5 (5.4)	0.42*	21 (1.8)	3 (3.2)	0.42*
BMI (kg/m^2^)												
<18.5	720 (69.5)	59 (54.1)		567 (47.9)	48 (48.0)		424 (36.8)	37 (39.8)		377 (33.2)	33 (35.5)	
18.5–24.9	445 (36.9)	44 (40.4)		583 (49.3)	42 (42.0)		686 (59.5)	48 (51.6)		713 (62.8)	51 (54.8)	
≥25	31 (2.6)	6 (5.5)	0.13*	33 (2.8)	10 (10.0)	0.002*	42 (3.6)	8 (8.6)	0.05*	46 (4.0)	9 (9.7)	0.04*
Mean (± SD)	18.05 (±2.94)	18.35(±3.72)	0.31	18.95 (±5.79)	21.29 (±20.46)	0.005	19.55 (±3.50)	19.98 (±3.86)	0.26*	19.78 (±2.87)	20.58 (±4.95)	0.02

*TB*: tuberculosis, *TBNDM*: TB without DM, *TBDM*: TB and DM, *BMI*: body mass index, *kg*: kilogram, *m* ^*2*^: meter square, *SD*: standared deviation

*Fisher’s exact test

^a^the number of total patients

**Table 4:** Sputum smear status of study participants at 2^nd^, 3^rd^, 5^th^ and 6^th^ months of anti-TB treatment period in South- Eastern Amhara Region, Ethiopia, September 2013-March 2015

Type of patients				
Sputum smear status	All TB n (%)	TBNDM n (%)	TBDM n (%)	*P*-value
2^nd^ month (*n* = 345)				
Positive	61 (17.7)	56 (17.6)	5 (18.5)	
Negative	284 (82.3)	262 (82.4)	22 (81.5)	1.00*
3^rd^ month (*n* = 63)				
Positive	22 (34.9)	19 (32.8)	3 (60.0)	
Negative	41 (65.1)	39 (67.2)	2 (40.0)	0.33*
5^th^ month (*n* = 324)				
Positive	9 (2.8)	9 (3.0)	-	
Negative	316 (97.2)	290 (97.0)	26 (100.0)	1.00*
6^th^ month (*n* = 318)				
Positive	1 (0.3)	1 (0.3)	-	
Negative	317 (99.7)	291 (99.7)	26 (100.0)	1.00*

*TB*: tuberculosis, *TBNDM*: TB without DM, *TBDM*: TB and DM

*Fisher’s exact test

**Table 5:** Factors associated with sputum smear conversion, September 2013-March 2015

Characteristics	Crude HR (95 % C.I.)	*P*-value	Adjusted HR (95 % C.I.)	*P*-value
Sex				
Male				
Female	0.98 (0.79–1.22)	0.88	0.99 (0.78–1.24)	0.96
Age in years				
15–50				
51–89	0.98 (0.67–1.44)	0.93	0.93 (0.63–1.38)	0.72
Residence				
Rural				
Urban	1.04 (0.82–1.31)	0.76	1.01 (0.79–1.28)	0.95
Type of patient				
TBNDM				
TBDM	0.95 (0.63–1.41)	0.78	0.97 (0.64–1.47)	0.90
HIV status				
Negative				
Positive	1.02 (0.78–1.34)	0.89	1.03 (0.78–1.35)	0.86
BMI baseline (kg/m^2^)				
< 18.5				
18.5–24.9	0.98 (0.77–1.24)	0.86	0.94 (0.73–1.21)	0.62
≥ 25	0.98 (0.46–2.08)	0.96	0.99 (0.46–2.13)	0.97
Adherence to TB				
Poor				
Good	2.92 (1.30–6.55)	0.009	3.13 (1.34-7.32)	0.009

*HR*: hazard ratio, *C.I.*: confidence interval, *TBNDM*: TB without DM, *TBDM*: TB and DM, *HIV*: human immuno deficiency virus, *BMI*: body mass index, *kg*: kilogram, *m* ^*2*^: meter square

### Treatment outcome

**Fig. 2:**
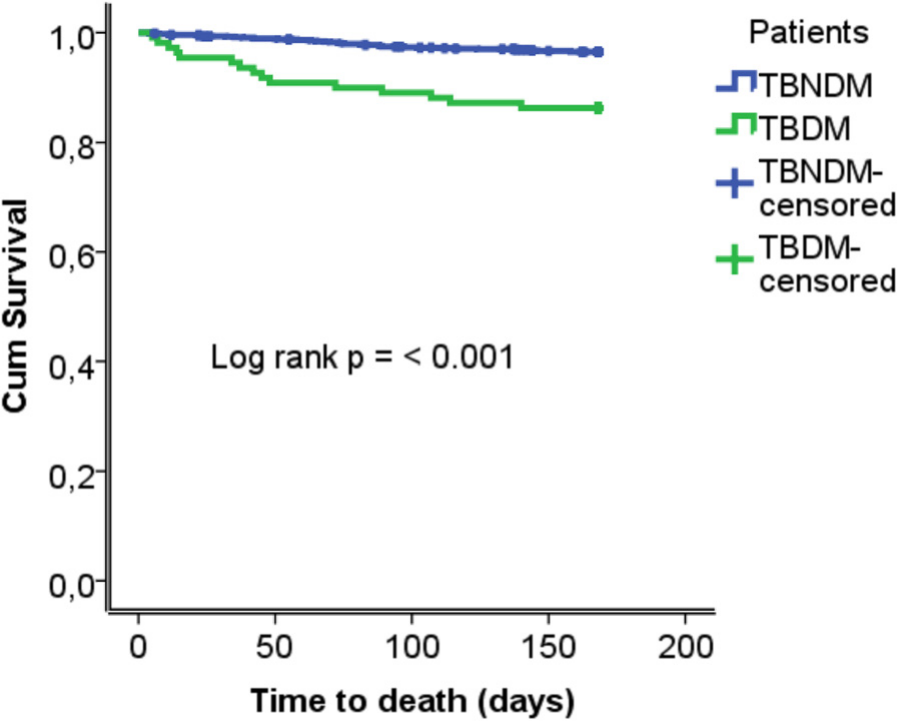
Kaplan-Meier curves for death comparing TBDM Vs. TBNDM patient groups

At the end of 6^th^ months anti-TB treatment period, 1135 (94.2 %) TBNDM and 93 (85.3 %) TBDM patients had successful treatment outcomes. The proportion of death 15 (13.8 %) observed in the TBDM patient group was higher compared to 42 (3.5 %) deaths seen in the TBNDM patient category (*p* < 0.001) (Fig. 2). Only one case of failure to treatment was observed in the TBDM group. A total of 1187 (98.5 %) patients in the TBNDM and 109 (100 %) participants in the TBDM patient group had good adherence to anti-TB treatment. Majority, 64 (58.7 %) of the TBDM patients had poor adherence to DM therapy (Table 6). In multivariate analysis, TBDM comorbidity [AHR 3.96; 95 % C.I., 1.76–8.89] and TBHIV coinfection [AHR 2.59; 95 % C.I., 1.21–5.59] were associated with increased death among patients (Table 7).

**Table 6:** TB treatment outcome of study participants, September 2013-March 2015

Characteristics	All TB n (%)	TBNDM n (%)	TBDM n (%)	*P*-value
Treatment outcome				
Cure^a^	317 (24.1)	291 (24.1)	26 (23.9)	0.95
Treatment completed^a^	911 (69.3)	844 (70.0)	67 (61.5)	0.06
Death^a^	57 (4.3)	42 (3.5)	15 (13.8)	<0.001
Treatment failure^a^	15 (1.1)	14 (1.2)	1 (0.9)	1.00*
Defaulter^a^	14 (1.1)	14 (1.2)	-	-
Total	1314 (100.0)	1205 (91.7)	109 (8.3)	
Over all treatment outcome (*n* = 1314)				
Successful	1228 (93.5)	1135 (94.2)	93 (85.3)	
Unsuccessful	86 (6.5)	70 (5.8)	16 (14.7)	<0.001
Adherence to TB treatment				
Poor	18 (1.4)	18 (1.5)	-	
Good	1296 (98.6)	1187 (98.5)	109 (100.0)	0.39
Adherence to DM therapy				
Poor	64 (58.7)	-	64 (58.7)	-
Good	45 (51.3)	-	45 (51.3)	

*TB*: tuberculosis, *TBNDM*: TB without DM, *TBDM*: TB and DM, *DM*: diabetes mellitus

*Fisher’s exact test

^a^Each outcome is compared to other treatment outcome category

**Table 7:** Factors associated with death among TB patients in South–Eastern Amhara Region, Ethiopia

Variables	Crude HR (95 % C.I.)	*P-*value	Adjusted HR (95 % C.I.)	*P*-value
Age in years				
15–50				
51–89	3.09 (1.80–5.29)	<0.001	2.40 (0.99–5.87)	0.05
Type of patient				
TBNDM				
TBDM	4.19 (2.33-7.57)	<0.001	3.96 (1.76–8.89)	0.001
Education				
Uneducated				
Educated	0.64 (0.38–1.09)	0.10	0.97 (0.42–2.23)	0.94
Sputum smear at baseline				
Negative				
Positive	0.46 (0.20–1.03)	0.06	0.57 (0.25–1.31)	0.18
HIV status				
Negative				
Positive	2.38 (1.39-4.08)	0.002	2.59 (1.21–5.59)	0.01

*HR*: hazard ratio, *C.I.*: confidence interval, *TB*: tuberculosis, *TBNDM*: TB without DM, *TBDM*: TB and DM, *HIV*: human immuno deficiency virus

## Discussion

In this study, we found no significant difference in clinical manifestations between TBDM and TBNDM patient groups except at the 2^nd^ month of treatment where patients in the TBDM group were more symptomatic compared to their counterparts. The overall successful treatment outcomes observed in both patient groups were good and exceeded the WHO target of achieving 85 % treatment success rate [23]. This may demonstrate an effective TB control program performance in the study area. It may also be an indication of active commitment of patients to their TB treatment. In addition, the result shows that the standard TB treatment regimen can be used for managing patients with TBDM comorbidity [13, 14].

There was a significant difference in treatment outcome between TBDM and TBNDM patient groups. Patients in the TBDM group were four times more likely to die compared to patients in the TBNDM category. This may be associated with poor glycemic control and impaired cell mediated immunity in TBDM patients [24–27]. The finding is in line with results of previous studies reported from Portugal [9], Maryland [11], Taiwan [24] and Malaysia [28]. On the other hand, studies conducted in Thailand and Fiji indicated that the rate of death was similar in both TBDM and TBNDM patient groups [14, 29]. The untoward effect of DM may have serious implications in meeting the 2035 target of achieving 95 % reduction in TB mortality as set by WHO [30]. Hence, our finding suggests a need for DM screening in TB patients. Screening TB patients for DM expedites early detection and treatment of patients with TBDM comorbidity. It may also enhance optimal glycemic control as part of the TBDM patient management [11, 14, 31, 32].

TB/HIV coinfected patients were more likely to die compared to HIV negative TB patients. HIV infection is a known risk factor for poor TB treatment outcome [33]. This finding suggests that there is a need to strengthen the existing TB/HIV collaborative activities in the study area.

At base line, TBDM and TBNDM patient groups did not show significant difference in clinical symptoms. The result is in agreement with the findings of several other studies done in Saudi Arabia [13], Thailand [14], Turkey [34], Tehran–Iran [35] and Tanzania [36], but is in contrast to the studies conducted in Texas-Mexico, Indonesia and Taiwan where TBDM patients at baseline were more symptomatic than their counterparts [7, 24, 32]. The reason for insignificant differences in the observed symptoms between the two patient groups in our study might be related to early health seeking and start of treatment among patients.

Weight loss, poor appetite and fatigue were the most frequent clinical manifestations seen at baseline in TBDM patients. This may indicate that symptoms of one disease resembles the other [1, 10, 25], and suggests the need for a high index of suspicion for TB and DM using bi-bidirectional screening approach for both diseases [31].

A high proportion of patients in the TBDM and TBNDM patient groups at baseline had a BMI value of <18.5 kg/m^2^. Evidence shows that there is a bidirectional causal link between undernutrition and active TB. Undernutrition in TB patient’s results in severe illness [37]. Undernutrition may also be a manifestation of poor glycemic control in diabetic TB patients [26]. Malnutrition stimulates the production of stress hormone which causes increased blood glucose level in TBDM patients [9]. Therefore, nutritional support and proper counseling is essential for patients with TBDM comorbidity [37].

During the course of anti-TB treatment, there was a significant increase in BMI among patients in the TBDM group compared to patients in the TBNDM category. This finding may be related to good recovery of TB illness among TBDM patients and signals the importance of adjusting drug dose based on patient’s weight [21]. Participants in the TBDM category were more symptomatic at the end of the intensive phase treatment period compared to patients in the TBNDM group. This finding is different from a previous observation, where symptomatic improvements were seen in both groups of patients at the 2^nd^ month of anti-TB treatment period [32]. The result may indicate delay in treatment response in TBDM patients and this in turn may be linked to poor glycemic control and lower concentrations of anti-TB drugs in plasma [24, 25, 38]. Close monitoring of blood glucose and clinical conditions of TBDM patients during the treatment period is crucial.

Both TBDM and TBNDM patient groups had better clinical improvements at 5^th^ and 6^th^ months of anti-TB chemotherapy compared to the baseline and 2^nd^ month of treatment. A similar finding was reported from Indonesia [32]. The result may indicate the effectiveness of the current anti-TB treatment regimen to manage patients with TBDM comorbidity.

Sputum smear conversions at the end of 2^nd^, 5^th^ and 6^th^ months of anti-TB treatment period were higher in both TBDM and TBNDM patient groups. The sputum conversion result at 2^nd^ month is consistent with the studies done in Maryland [11], Thailand [14] and Fiji [29], but is different from the findings of studies conducted in Texas-Mexico [7], Taiwan [8], Maharashtra-India [10], Saudi Arabia [13], Taiwan [24] and Turkey [34] where sputum conversion among TBDM patient groups were lower. The good sputum smear conversions observed in both patient groups in our study may be related to good treatment adherence among patients. Poor adherence to anti-TB treatment is associated with sub-therapeutic levels of anti-TB drugs and often results in treatment failure. In addition, poor adherence to treatment is a driving force for the emergence and spread of drug-resistant TB [39, 40].

In this study, no defaulter and only one treatment failure case was observed in the TBDM patient group compared to a relatively higher number of defaulters and treatment failure cases observed in TBNDM patient category. Studies conducted in Taiwan, Maryland, Thailand and Indonesia [8, 11, 14, 32] showed that the risk of treatment failure was higher in TBDM patients than patients in the TBNDM category. The absence of defaulters in the TBDM patient group is different from the study done in Thailand [14]. High default rate is a challenge for successful TB control program performance. It is associated with poor access to HFs, adverse drug reactions, social stigma and lack of awareness about the consequences of TB disease [41]. Several reasons including the effective TB control program implementation in the study area, and good treatment adherence among patients may have contributed to the low number of treatment failure and defaulter cases observed in both patient groups.

This study has several strengths. To the best of our knowledge, this is one of the very few studies conducted in Africa and may be used as a baseline for future larger studies. The study was conducted at all levels of government and private HFs. A large number of study participants selected from urban and rural areas were enrolled in the study. All these minimize selection bias. In addition, the study employed cohort study design. Patients were prospectively followed, clinical characteristics and treatment outcomes were properly documented and findings were compared in both TBDM and TBNDM patient groups. Moreover, information bias due to patient transfer out and lost to follow-up were very well controlled by setting proper inclusion criteria and conducting strict patient follow-up during the entire treatment period.

The study has potential limitations. Due to lack of advanced laboratory facilities for performing all of the necessary laboratory investigations for screening and patient follow-up, comparison of findings between TBDM and TBNDM patient groups were based on routine biochemical and microscopic test results. There was poor blood glucose level follow-up in TBDM patients. Hence, it was not possible to assess the role of blood glucose level on clinical manifestations and mortality among patients with TBDM comorbidity.

## Conclusion

The study showed that DM is associated with increased mortality during TB treatment. The result also demonstrated that there is no difference in clinical presentations and bacteriological findings in both TBDM and TBNDM patient groups at baseline and during anti-TB treatment follow-up period. However, at the 2^nd^ month of treatment, TBDM patients were more symptomatic compared to patients in the TBNDM group. In order to expedite early diagnosis and treatment of patients with TBDM comorbidity, we recommend routine screening of DM in TB patients in the study area.

### Data availability

The data are presented in the main paper and additional supporting file (Additional file 2).

## Additional files

Additional file 1: Multilingual abstracts in the six official working languages of the United Nations. (PDF 297 kb) Additional file 2: Dataset used for the manuscript. (XLS 3953 kb)

## Abbreviations

AFB: acid fast bacilli
AHR: adjusted hazard ratio
ART: anti-retroviral therapy
BMI: body mass index
C.I.: confidence interval
DM: diabetes mellitus
DOTS: directly observed treatment short course
EPTB: extra pulmonary tuberculosis
FBS: fasting blood sugar
HFs: health facilities
HIV: human immune-deficiency virus
MDG: Millennium Development Goal
MDR: multi-drug resistant
PICT: provider initiated counseling and testing
PTB: pulmonary tuberculosis
RBS: random blood sugar
SD: standard deviation
SPSS: statistical package for social science
TB: tuberculosis
TBDM: tuberculosis and diabetes mellitus
TB/HIV: tuberculosis and human immune-deficiency virus
TBNDM: tuberculosis without diabetes mellitus
WHO: World Health Organization
USD: United State Dollar
