# Supplementary material for: Diabetes mellitus is associated with increased mortality during tuberculosis treatment: a prospective cohort study among tuberculosis patients in South-Eastern Amahra Region, Ethiopia
Source: Infect Dis Poverty. 2016 Mar 21;5:22. doi: 10.1186/s40249-016-0115-z (PMC4806519; doi:10.1186/s40249-016-0115-z)
Supplement: Additional file 1: — Multilingual abstracts in the six official working languages of the United Nations. (PDF 297 kb) [file 40249_2016_115_MOESM1_ESM.pdf]

Translation of the abstract into the five official working languages of the United Nations

يصاحب مرض السكري زيادة في معدل الوفيات أثناء علاج مرض السل: دراسة تعرض مستقبلية بين مرضى السل في منطقة الأمهرا جنوب شرق إثيوبيا.

Solomon Abebe Yimer , Mahteme Haile Workneh, Gunnar Aksel Bjune

موجز

تمهيد: هناك تزايد في عدد الأدلة التي تشير إلى أن مرض السكري يؤثر على تجليات المرض ونتائج العلاج عند مرضى السل.هدف هذه الدراسة هو التعرف على تأثير مرض السل على التجليات السريرية والنتائج العلاجية بين مرضى السل المشخصين حديثا.

الأدوات: أ . أجريت دراسة تعرض مستقبلية في أمهرا جنوب شرق إثيوبيا من سبتمبر 2013 إلى غاية مارس 2015. تم إختيارالأشخاص الذين سيخضعون للدراسة بشكل عشوائي من 44 مرفقا صحيا على التوالي موجودة في المنطقة المستهدفة من الدراسة. صنف المشاركون إلى نوعين من المرضى في مجموتين ، المرضى المصابون بالسل و مرض السكري (TBDM) و مرضى السل الغير مصابين بمرض السكري (TBDNM). وتمت مقارنة التجليات السريرية والنتائج العلاجية بين المجموعتين .وبعد ذلك تم تطبيق نموذج إنحدار كوكس التحليلي لتحديد العوامل المرتبطة بالوفيات.

النتائج: من بين 1314 مريض بالسل مسجل في الدراسة، 109 أو (8.3٪) من المرضى مصابون بمرض السكري من النوع الذي يمكن التعايش معه. الإصابة المرضية المشتركة للمصابين بمرض السل و مرض السكري [نسبة الخطر المعدلة (AHR) 3.96. نطاق الثقة 95٪ (CI) (1،76-8،89)]،تم ربط عدوى السل المصحوبة بفيروس نقص المناعة البشرية بزيادة احتمال الوفاة (HIV) [AHR 2.59. 95٪ CI (1،21-5،59)] . ليس هناك فرق كبيرفي الأعراض السريرية في بداية أو خلال فترة العلاج ضد للسل بين المرضى المصابون بالسل و مرض السكري (TBDM) من جهة و مرضى السل الغير مصابين بمرض السكري(TBDNM) من جهة أخرى.|

الاستنتاجات: أظهرت الدراسة أن مرض السكري مرتبط بزايدة احتمال الوفاة أثناء علاج السل. ليس لمرض السكري أي ارتباط بالتجليات السريرية لمرض السل باستثناء في نهاية مرحلة العلاج المكثف. ينصح الفحص الروتيني لمرضى السل للتأكد من عدم الإصابة بمرض السكرى للتشخيص المبكر وعلاج المرضى الذين يعانون من الاصابة المشتركة بمرض السكري و مرض السل(TBDM).

Translated from English version into Arabic by Azeez Hamdoun, through

TRANSLATORS
WITHOUT BORDERS

糖尿病增加结核病治疗中的死亡：在埃塞俄比亚阿姆哈拉州东南部的结核病患者前瞻性队列研究

Mahteme Haile Workneh, Gunnar Aksel Bjune, Solomon Abebe Yimer

摘要

引言：越来越多的证据表明，糖尿病（DM）影响结核病（TB）患者的疾病表现和治疗结果。本研究旨在调查DM对新诊断TB患者的临床表现和治疗结果的影响。

方法：2013年9月至2015年3月，在埃塞俄比亚阿姆哈拉州东南部开展了一项前瞻性队列研究。从研究区域中随机选取的44家卫生机构中连续招募研究对象。研究对象分为两组，分别是有糖尿病的结核病患者（TBDM）和无糖尿病的结核病患者（TBNDM）。比较两组患者的临床表现和治疗效果。应用Cox比例风险回归分析确定死亡相关因素。

结果：在1 314例纳入研究的TB患者中，109例（8.3%）合并DM。TB伴有DM及TB伴有人类免疫缺陷病毒（HIV）均会增加死亡风险，前者校正风险比（AHR）为3.96（95％置信区间为1.76～8.89），后者AHR为

2.59（95％置信区间为1.21～5.59）。TBDM和TBNDM患者的临床症状在基线和抗结核治疗期间的差异均无统计学意义。但在治疗的第2个月，TBDM患者组较TBNDM患者组症状更明显。

结论：研究表明DM与TB治疗过程中的死亡升高有关。除强化治疗阶段外，DM与TB的临床表现无关。建议对TB患者进行DM常规筛查以对TBMD合并症患者尽早诊断和治疗。

Translated from English version into Chinese by Qian Menbao, edited by Yang Pin, through

CHINESE CENTER FOR DISEASE CONTROL AND PREVENTION
NATIONAL INSTITUTE OF PARASITIC DISEASES

Association du diabète sucré à une hausse de la mortalité au cours du traitement de la tuberculose : une étude de cohortes prospective menée parmi des patients atteints de tuberculose dans le sud-est de la région Amhara, Éthiopie

Mahteme Haile Workneh, Gunnar Aksel Bjune, Solomon Abebe Yimer

Résumé

Contexte : il apparaît de plus en plus clairement que le diabète sucré (DM) affecte la présentation pathologique et le résultat thérapeutique des patients atteints de tuberculose (TB). Cette étude a été menée afin d'examiner le rôle du DM sur les présentations cliniques et les résultats thérapeutiques parmi des patients chez lesquels la tuberculose a récemment été diagnostiquée.

Méthodes : une étude de cohortes prospective a été menée dans le sud-est de la région Amhara (Éthiopie) de septembre 2013 à mars 2015. Les sujets d'étude ont été consécutivement recrutés à partir de 44 installations de santé sélectionnées de manière aléatoire dans la région d'étude. Les participants ont été classés en deux groupes de patients : d'une part les patients atteints de tuberculose (TB) et de DM (TBDM) et d'autre part les patients atteints de TB mais pas de DM (TBNDM). Les observations faites sur les présentations cliniques et les résultats thérapeutiques ont été comparées entre les deux groupes de patients. Une analyse de régression des risques proportionnels de Cox a été réalisée pour identifier des facteurs associés au décès.

Résultats : sur 1314 patients atteints de TB recrutés dans le cadre de cette étude, 109 personnes (8,3 %) présentaient un DM coexistant. La comorbidité TBDM [risque relatif ajusté (RRA) 3,96 ; intervalle de confiance (IC) à 95 % (1,76-8,89)] et la co-infection TB/virus de l'immunodéficience humaine (VIH) [RRA 2,59 ; IC à 95 % (1,21-5,59)] étaient associées à une hausse des décès. Les patients TBDM et TBNDM ne présentaient aucune différence significative en termes de symptômes cliniques ni à la référence ni durant la période de traitement anti-TB. Néanmoins, les patients TBDM étaient plus symptomatiques par rapport aux patients du groupe TBNDM au deuxième mois de traitement.

Conclusions : l'étude a démontré que le DM est associé à une hausse des décès au cours du traitement de la TB. Le DM ne présente aucune association avec la présentation clinique de la TB, sauf à la fin de la phase de traitement intensif. Le dépistage de routine du DM chez des patients atteints de TB est recommandé pour le diagnostic et le traitement précoces de patients atteints d'une comorbidité TBDM.

Translated from English version into French by eric ragu, through

Взаимосвязь сахарного диабета с увеличением смертности в процессе лечения туберкулеза: Проспективное групповое исследование пациентов, больных туберкулезом, проживающих в регионе Юго-восточная Амхара, Эфиопия

Матем Хайле Воркне, Гуннар Аксель Бьюн, Соломон Абебе Йимер (Mahteme Haile Workneh, Gunnar Aksel Bjune, Solomon Abebe Yimer)

Отрывок

История вопроса: Все большее количество фактов свидетельствуют о том, что сахарный диабет оказывает влияние на проявление заболевания и результаты лечения у пациентов, больных туберкулезом. Целью данного исследования является изучение роли сахарного диабета в клинических картинах и результатах лечения у пациентов с недавно диагностированным туберкулезом.

Методы: В период с сентября 2013 г. по март 2015 г. в регионе Юго-восточной Амхары, в Эфиопии, проводилось проспективное групповое исследование. Субъекты исследования последовательно отбирались из 44 случайно выбранных медицинских учреждений в районе исследования. Участники были разделены на две группы пациентов, а именно, пациентов, больных туберкулезом наряду с сахарным диабетом, и пациентов, больных туберкулезом, но не больных сахарным диабетом. Затем было произведено сравнение полученных результатов клинических проявлений и результатов лечения двух групп пациентов. Для выявления факторов, связанных с летальным исходом, использовалась модель пропорциональных рисков Кокса.

Результаты: У 109 (8,3%) из 1314 включенных в исследование пациентов, больных туберкулезом, был выявлен сопутствующий сахарный диабет. Была выявлена взаимосвязь между сопутствующим заболеванием сахарным диабетом [скорректированное отношение рисков (СОР) 3,96; доверительный интервал (Д.И.) 95% (1,76-8,89)], а также ассоциированной инфекцией туберкулеза с вирусом иммунодефицита человека (ВИЧ) [СОР 2,59; Д.И. 95% (1,21-5,59)] и повышенной смертностью. Клинические симптомы у пациентов, больных наряду с туберкулезом сахарным диабетом, и не больных ассоциированным с туберкулезом сахарным диабетом, на исходном уровне и в ходе лечения туберкулеза не демонстрировали сильных различий. Однако, по окончании $2^{го}$ месяца лечения, пациенты, больные ассоциированным с туберкулезом сахарным диабетом, демонстрировали больше клинических проявлений, по сравнению с группой пациентов, не больных ассоциированным с туберкулезом сахарным диабетом.

Заключение: Исследование продемонстрировало взаимосвязь повышенной смертности с наличием заболевания сахарным диабетом в ходе лечения туберкулеза. Взаимосвязь сахарного диабета с клиническими проявлениями туберкулеза проявилась лишь в конце интенсивной фазы лечения. Рекомендуется проводить плановое обследование пациентов, больных туберкулезом, на наличие сахарного диабета, для раннего распознавания болезни и лечения пациентов, больных туберкулезом с сопутствующим сахарным диабетом.

Translated from English version into Russian by tatiana_com, through

La diabetes mellitus está relacionada con el aumento de la mortalidad durante el tratamiento de la tuberculosis: estudio de cohorte prospectivo en pacientes de tuberculosis en la región sudoriental de Amhara en Etiopía.

Mahteme Haile Workneh, Gunnar Aksel Bjune, Solomon Abebe Yimer

Resumen

Introducción: La creciente evidencia indica que la diabetes mellitus (DM) afecta el cuadro clínico y los resultados del tratamiento en pacientes con tuberculosis (TB). El objetivo del estudio es investigar el papel de la DM en cuadros clínicos y los resultados del tratamiento en pacientes a los que se les acaba de diagnosticar TB.

Métodos: Se llevó a cabo un estudio de cohorte prospectivo en la región sudoriental de Amhara en Etiopía, entre septiembre del 2013 y marzo del 2015. Los participantes fueron reclutados consecutivamente en 44 centros de salud seleccionados al azar en la región. Los mismos fueron divididos en dos grupos de pacientes; es decir, uno de pacientes con TB y DB (TBDM) y otro de pacientes con TB sin DB (TBNDM). Se compararon los resultados de los cuadros clínicos y los resultados del tratamiento, entre los dos grupos de pacientes y se utilizó el análisis de regresión de riesgos proporcionales de Cox para identificar los factores relacionados con la mortalidad.

Resultados: 109 (8,3%) de los 1314 pacientes con TB que formaron parte del estudio tenían DM concomitante. La comorbilidad TBDM [índice de riesgo ajustado (IRA) de 3,96; 95% de intervalo de confianza (IC) (1,76-8,89)] y la coinfección de TB con el virus de inmunodeficiencia humana (VIH) [IRA de 2,59; 95% de CI. (1,21-5,59) estaban relacionados con el aumento en la mortalidad. Los pacientes con TBDM y TBNDM no mostraron una diferencia significativa de síntomas clínicos al inicio y durante el periodo de tratamiento anti-TB. Sin embargo, durante el 2° mes de tratamiento, los pacientes con TBDM presentaron más síntomas en comparación con los pacientes en el grupo TBNDM.

Conclusiones: El estudio demostró que la DM está relacionada con el aumento de la mortalidad durante el tratamiento de TB. La DM no tiene relación con el cuadro clínico de TB, excepto al final de la fase intensiva del tratamiento. Se recomienda realizar estudios de rutina para detectar la DM en pacientes con TB para lograr un diagnóstico y tratamiento precoz en pacientes con comorbilidad TBDM.

Translated from English version into Spanish by Sión Guitart, through
